# Peripheral artery disease in pseudoxanthoma elasticum: Walking capacity on the 6-minute walking test

**DOI:** 10.1177/1358863X261434827

**Published:** 2026-06-11

**Authors:** Melanie Haverkamp, Frank LJ Visseren, Constantijn EVB Hazenberg, Pim A de Jong, Wilko Spiering

**Affiliations:** 1 Department of Vascular Medicine and Endocrinology, University Medical Center Utrecht, Utrecht University, Utrecht, The Netherlands; 2 Department of Vascular Surgery, University Medical Center Utrecht, Utrecht University, Utrecht, The Netherlands; 3 Department of Radiology, University Medical Center Utrecht, Utrecht University, Utrecht, The Netherlands

**Keywords:** peripheral artery disease (PAD), pseudoxanthoma elasticum (PXE), treadmill test, WELCH questionnaire

Pseudoxanthoma elasticum (PXE) is a multisystemic disease caused by pathogenic variants in the *ABCC6* gene, resulting in ectopic calcifications. PXE leads to severe vision loss, skin abnormalities, and the risk of cerebrovascular events and peripheral artery disease (PAD).^1^ In patients with PXE, arterial calcifications are primarily present in peripheral arteries.^1^ Calcifications in these arteries may cause obstructive PAD, which affects more than 50% of patients with PXE.^2^ PAD is more prevalent in PXE than in the general population of high-income countries (⩾ 50% vs 4%) and also occurs at younger ages than in the general population.^2,3^ PAD results in claudication and nocturnal pain, which impacts patients’ quality of life. As PAD occurs at younger ages in PXE, we hypothesized that walking distances (WDs) in PXE would be reduced. Therefore, we aimed to evaluate functional capacity in patients with PXE compared with healthy references using the 6-minute walking test (6MWT).

Patients originated from the Dutch UMC Utrecht Expertise Center for PXE (UECP) and are participating in the Dutch PXE Registry. This study was approved by the institutional review board of the UMC Utrecht (reference number: 18-767/M). All patients gave their written informed consent. Patients were included from February 2022, when the 6MWT became part of standard care. Patients were diagnosed with PAD if the rest ankle–brachial index (ABI) was ⩽ 0.9 or if the rest ABI was normal but decreased (> 20%) after a treadmill test. During routine visits, patients underwent vascular examinations, including a 6MWT. WDs were recorded, and reference values were calculated using standard equations.^4^ Differences between the WDs of patients with PXE and their healthy reference values were compared with a paired *t*-test. Observed and reference WDs were stratified by sex, PAD, and age groups. Detailed information regarding study methods and analysis is provided in the supplemental material.

In total, 162 patients were included, with a mean age of 50 ± 14 years, of whom 52% were women (Supplemental Table S1). Among these patients, 18% reported claudication and 27% reported nocturnal leg cramps. The mean observed WD in women with PXE was 533 ± 96 meters and was significantly lower than reference values (–32 m; 95% CI: −50 to −13 m). Similarly, men walked significantly shorter distances than expected (–69 m; 95% CI: −93 to −44 m). Among younger women and men (< 50 years), the WD was significantly lower than their reference values (women: −66 m; 95% CI: −89 to −42 m; men: −102 m; 95% CI: −148 to −69 m) (Figure 1). In total, 111 patients with PXE (69%) had a lower WD compared with their healthy references. Ninety-two patients underwent additional pre- and postexercise ABI measurements, and 52 were diagnosed with PAD. PAD was present in 47% of patients < 50 years and 61% of those aged ⩾ 50 years. Among patients with PXE and PAD (*n* = 52), WD (496 ± 109 m) was significantly below reference values (–60 m; 95% CI: −89 to −32 m) (Figure 1). In this group, 36 patients (69%) had a lower WD compared to their healthy references. Among patients without PAD (*n* = 40), the WD (554 ± 79 m) was also significantly less than their reference values (–53 m; 95% CI: −87 to −19 m). Twenty-two patients with PXE and without PAD (55%) had a lower WD compared to their healthy references.

**Figure 1.**
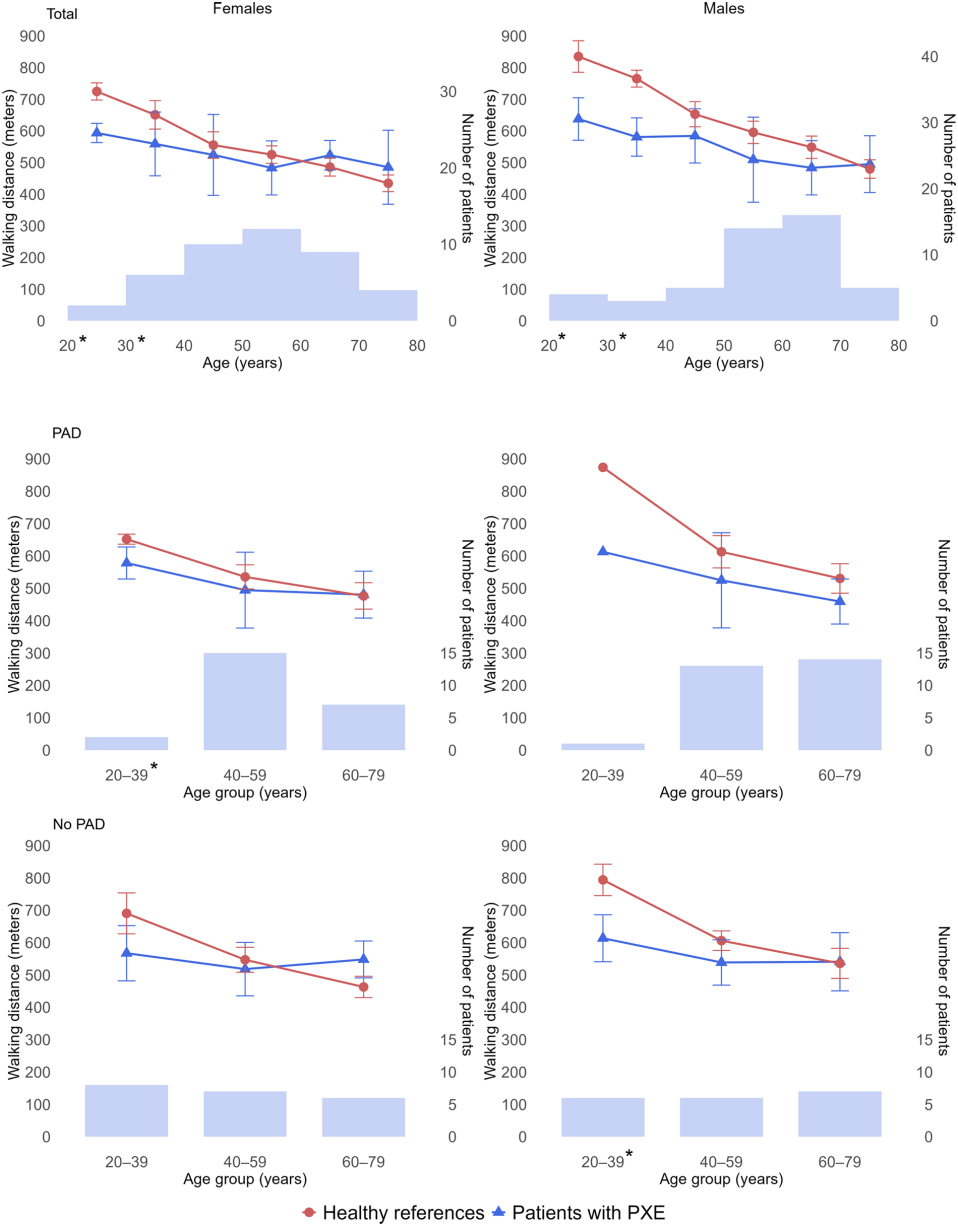
Walking distance across age categories in male and female patients with PXE compared with healthy reference values. Blue triangles represent observed mean walking distance in patients with PXE, and red circles represent expected mean walking distance based on healthy reference data. Error bars indicate standard deviations. The light blue bars represent the number of patients within each age category. Asterisk (*) indicates a significant difference (p < 0.05).

This study found that walking capacity in PXE was reduced, particularly in younger patients, regardless of PAD status. We hypothesized several possible explanations for this. First, vascular involvement in PXE occurs at young ages, resulting in early onset of claudication symptoms, fatigue, and reduced walking capacity. In contrast, young, healthy references typically experience no limitations, resulting in a larger gap. Second, in older age groups, the difference may be attenuated due to a natural decline in WDs and more comorbidities among healthy reference populations. Third, the reference equations were derived from healthy adults aged ⩾ 40 years. For individuals below 40 years, the healthy reference values are extrapolated and may overestimate walking performance.

WDs in healthy populations range from 494 to 555 meters in women and 576 to 585 meters in men.^4,5^ The difference in WD between healthy populations and patients with PXE seems relatively small. However, these healthy WDs are not adjusted for age and body mass index (BMI), which can significantly influence walking performance and make it hard to compare these populations.^4^ Lower WDs are also observed in non-PXE patients with PAD. Studies about PAD report WDs ranging from 324 to 360 meters.^6–8^ These distances are lower than distances observed in our study, which is likely due to PAD severity. Our patients with PAD had a resting ABI of 0.74, whereas patients in the other studies had a mean resting ABI ranging from 0.58 to 0.65.^6–8^ Reduced WD in PAD is associated with cardiovascular disease, mobility loss, and mortality.^8–10^ Although not investigated in PXE, its presence in PAD may suggest a possible relationship, highlighting the need to address the consequences of early walking decline in PXE.

The strengths of this study are the relatively large cohort for a rare disease and the first walking capacity assessment in PXE. A limitation is that PAD status was available for only 92 of 162 patients, although this subset was representative of the total cohort (Table S2).

In conclusion, this study shows that the walking capacity of patients with PXE is less than that of their healthy references and is independent of PAD. Longitudinal studies are needed to investigate the relationship between reduced walking capacity and cardiovascular risk in PXE.

## Supplemental Material

sj-docx-1-vmj-10.1177_1358863X261434827 – Supplemental material for Peripheral artery disease in pseudoxanthoma elasticum: Walking capacity on the 6-minute walking test Supplemental material, sj-docx-1-vmj-10.1177_1358863X261434827 for Peripheral artery disease in pseudoxanthoma elasticum: Walking capacity on the 6-minute walking test by Melanie Haverkamp, Frank LJ Visseren, Constantijn EVB Hazenberg, Pim A de Jong and Wilko Spiering in Vascular Medicine
