# Supplementary material for: Peripheral artery disease in pseudoxanthoma elasticum: Walking capacity on the 6-minute walking test
Source: Vasc Med. 2026 Jun 11;31(4):562–4. doi: 10.1177/1358863X261434827 (PMC13451603; doi:10.1177/1358863X261434827)
Supplement: Supplementary file 1 [file sj-docx-1-vmj-10.1177_1358863X261434827.docx]

SUPPLEMENTAL DATA

TABLES

| Table S1: Baseline characteristics of patients with PXE | | |
| --- | --- | --- |
|  | Total  N = 162 | Patients with ABI measurements  N = 92 |
| Age (years) | 50 ± 14 | 53 ± 14 |
| Sex (male) | 77 (48) | 47 (51) |
| Smoking status |  |  |
| Never | 67 (42) | 33 (36) |
| Former | 78 (48) | 49 (54) |
| Current | 16 (10) | 9 (10) |
| Diabetes mellitus | 16 (10) | 14 (15) |
| History of CVD |  |  |
| CAD | 2 (1) | 1 (1) |
| CeVD | 20 (13) | 15 (17) |
| PAI | 1 (1) | 1 (1) |
| claudication* | 29 (18) | 22 (24) |
| Cramps legs* | 43 (27) | 31 (34) |
| WELCH score* | 72 [54;80] | 70 [45;80] |
| Antihypertensive therapy | 39 (24) | 21 (23) |
| Lipid-lowering therapy | 61 (38) | 41 (45) |
| Antithrombotic therapy | 27 (17) | 19 (21) |
| SBP (mmHg)* | 128 ± 19 | 129 ± 19 |
| BMI (kg/m²) | 27 ± 5.2 | 27.5 ± 5.0 |
| Resting ABI* | 0.92 [0.71;1.00] | 0.90 [0.74;0.99] |
| Resting ABI >1.4 | 0 (0) | 0 (0) |
| Treadmill ABI* | 0.88 [0.63;1.01] | 0.88 [0.63;1.01] |
| Pre 6MWT SpO_2_ | 99 [97;99] | 98 [97;99] |
| Post 6MWT SpO_2_ | 98 [97;99] | 98 [97;99] |
| Pre-6MWT heart rate | 75 ± 12 | 76 ± 14 |
| Post 6MWT heart rate | 106 ± 23 | 103 ± 24 |
| Total cholesterol (mmol/L)* | 4.5 ± 0.9 | 4.6 ± 1.0 |
| LDL-cholesterol (mmol/L)* | 2.5 ± 0.8 | 2.5 ± 0.9 |
| HDL-cholesterol (mmol/L)* | 1.5 ± 0.4 | 1.4 ± 0.4 |
| VLDL-cholesterol (mmol/L)* | 0.5 [0.3;0.7] | 0.5 [0.3;0.7] |
| eGFR (mL/min/1.73m2)* | 96 ± 18 | 94 ± 19 |

Data are presented as numbers (%), mean ± standard deviation, or median [interquartile range]. Abbreviations: CVD: cardiovascular disease; CAD: coronary artery disease; CeVD: cerebrovascular disease; PAI: peripheral artery disease with arterial interventions; SBP: systolic blood pressure; BMI: body mass index; ABI: ankle-brachial index; 6MWT: six-minute walking test; LDL: low-density lipoprotein; HDL: high-density lipoprotein; VLDL: very low-density lipoprotein; eGFR: estimated glomerular filtration rate. *Data was not available for all patients

| Table S2: Comparison of baseline characteristics between included patients and the total PXE Registry | | | |
| --- | --- | --- | --- |
|  | Patients in the current study  N = 162 | Patients with ABI measurements  N = 92 | Patients in the total PXE Registry  N = 247 |
| Age (years) | 50 ± 14 | 53 ± 14 | 54 ± 14 |
| Sex (male) | 77 (48) | 47 (51) | 91 (37) |
| Smoking status |  |  |  |
| Never | 67 (42) | 33 (36) | 90 (36) |
| Former | 78 (48) | 49 (54) | 119 (48) |
| Current | 16 (10) | 9 (10) | 17 (15) |
| Diabetes mellitus | 16 (10) | 14 (15) | 18 (7) |
| History of CVD | 22 (14) | 17 (18) | 28 (11) |
| Antihypertensive therapy | 39 (24) | 21 (23) | 73 (30) |
| Lipid-lowering therapy | 61 (38) | 41 (45) | 89 (36) |
| Antithrombotic therapy | 27 (17) | 19 (21) | 55 (22) |
| SBP (mmHg)* | 128 ± 19 | 129 ± 19 | 135 ± 20 |
| BMI (kg/m²) | 27.0 ± 5.2 | 27.5 ± 5.0 | 26.2 ± 4.8 |
| Total cholesterol (mmol/L)* | 4.5 ± 0.9 | 4.6 ± 1.0 | 5.1 ± 1.2 |
| LDL-cholesterol (mmol/L)* | 2.5 ± 0.8 | 2.5 ± 0.9 | 3.0 ± 1.0 |
| HDL-cholesterol (mmol/L)* | 1.5 ± 0.4 | 1.4 ± 0.4 | 1.5 ± 0.4 |
| VLDL-cholesterol (mmol/L)* | 0.5 [0.3;0.7] | 0.5 [0.3;0.7] | 0.5 [0.4;0.8] |
| eGFR (mL/min/1.73m2)* | 96 ± 18 | 94 ± 19 | 94 ± 18 |

Data are presented as numbers (%), mean ± standard deviation, or median [interquartile range]. Abbreviations: CVD: cardiovascular disease including coronary artery disease and cerebrovascular disease; SBP: systolic blood pressure; BMI: body mass index; LDL: low-density lipoprotein; HDL: high-density lipoprotein; VLDL: very low-density lipoprotein; eGFR: estimated glomerular filtration rate. *Data was not available for all patient

SUPPLEMENTAL METHODS

The Dutch PXE Registry uses pseudonymized data from regular care visits. This study is approved by the institutional review board of the UMC Utrecht (reference number: 18-767/M). Patients with a probable or definitive PXE diagnosis, as defined by the Plomp criteria, and who had completed at least one 6MWT were included in this study.^1^ The 6MWT has been part of standard care since 2022, and therefore, not all patients in the Dutch PXE Registry have completed a 6MWT so far.

At regular care visits, data were collected on medication use, medical history, smoking, self-reported maximal walking distances (MWD), and experience of nocturnal cramps in the legs. The WELCH questionnaire was asked during a clinic visit and consists of four questions assessing walking duration at different paces: slow, normal, and quick, and the patient is asked to compare his/her walking speed with the walking speed of their relatives or other people of the same age. The WELCH score ranges from 0 to 100 and is calculated as previously described.^2^ A score of 0 indicates a patient who can only walk slowly for 30 seconds, and a score of 100 indicates a patient who can walk fast for more than 3 hours. During physical examination, blood pressure was measured, and laboratory measurements were retrieved. The systolic and diastolic blood pressures are the mean of three consecutive measurements. The low-density lipoprotein cholesterol (LDL-c) levels were calculated according to the Friedewald formula.^3^ The estimated glomerular filtration rate (eGFR) was calculated using the CKD-EPI formula.^4^ Diabetes mellitus (DM) was defined as a confirmed DM diagnosis in the medical history or the use of glucose-lowering therapy during the 6MWT.

*Vascular measurements*

Patients also underwent vascular examinations, including ABI measurement, treadmill test (TT), and a 6MWT. The 6MWT was performed in a large exercise room under the supervision of a physiotherapist. Patients were instructed to walk as far as possible in six minutes, according to standardized instructions.^5^ The MWD is recorded after the test. Oxygen saturation, heart rate, and the Borg scores for dyspnea and fatigue (both general fatigue and fatigue of the legs) are recorded before and after the test. ^6^ Oxygen saturation was measured at the index finger using a pulse oximeter. Walking distances for healthy references were calculated based on age, sex, and BMI using standardized equations.^7^ The TT is performed for five minutes on a treadmill with a speed of 3.5 km/hour and a 10% incline. The ABI is measured by trained research nurses, before and one minute after the test. If the ABI was lower than the ABI at rest, the ABI measurements were repeated after three, five, and ten minutes. The lower ABI value of the left or right leg was used for the diagnosis of PAD.

*Data analysis*

Baseline characteristics are presented as counts and percentages for categorical variables, mean ± standard deviation for normally distributed variables, and median with interquartile range for non-normally distributed variables. The differences between the walking distances of patients with PXE and their expected healthy reference walking distances were compared using a paired t-test. Additionally, a stratified analysis according to age, PAD diagnosis, and sex was performed to assess the difference between younger and older patients with PXE and between males and females. To visualize the difference between sex and age, mean values and standard deviations of observed and expected reference walking distances were calculated and plotted against age groups (>20 years, 20-29 years, 30-39 years, 40-49 years, 50-59 years, 60-69 years, and 70-80 years). A two-sided α of 0.05 was considered statistically significant. All analyses were conducted with R statistical software version 4.4.2.

SUPPLEMENTAL REFERENCES

1. Plomp AS, Toonstra J, Bergen AAB, Van Dijk MR, De Jong PTVM. Proposal for updating the pseudoxanthoma elasticum classification system and a review of the clinical findings. *Am J Med Genet A*. 2010;152:1049–1058.

2. Abraham P, Godet R, Harbonnier M, Laneelle D, Leftheriotis G, Ouedraogo N. External validation of the “walking estimated limitation calculated by history” (WELCH) questionnaire in patients with claudication. *Eur J Vasc Endovasc Surg*. 2014;47:319–25.

3. Friedewald WT, Levy RI, Fredrickson DS. Estimation of the concentration of low-density lipoprotein cholesterol in plasma, without use of the preparative ultracentrifuge. *Clin Chem*. 1972;18:499–502.

4. Levey AS, Stevens LA, Schmid CH, et al. A New Equation to Estimate Glomerular Filtration Rate. *Ann Intern Med*. 2009;150:604.

5. ATS Committee on Proficiency Standards for Clinical Pulmonary Function Laboratories. ATS statement: guidelines for the six-minute walk test. *Am J Respir Crit Care Med*. 2002;166:111–7.

6. Borg GA. Psychophysical bases of perceived exertion. *Med Sci Sports Exerc*. 1982;14:377–81.

7. Enright PL, Sherrill DL. Reference equations for the six-minute walk in healthy adults. *Am J Respir Crit Care Med*. 1998;158:1384–7.
